# Burkitt’s Lymphoma of the Colon: A Case Report and Review of the Texas Cancer Registry

**DOI:** 10.7759/cureus.27964

**Published:** 2022-08-13

**Authors:** Yuichiro Z Sato, Rivers A Hock, Roberto L Garcia, Fatma Dihowm

**Affiliations:** 1 Geriatrics, Kaiser Permanente Medicine, San Fransisco, USA; 2 Internal Medicine, Texas Tech University Health Sciences Center, El Paso, USA; 3 Internal Medicine, University of Texas Health Science Center at Houston, Houston, USA

**Keywords:** texas cancer registry, case report, irritable bowel syndrome, colon, burkitt lymphoma

## Abstract

Burkitt’s lymphoma (BL) is an aggressive form of non-Hodgkin’s B-cell lymphoma with gastrointestinal (GI) involvement, but very few cases report primary colonic findings. We report one case of primary sporadic BL of the colon with non-specific GI symptoms, and its morphologic and immunohistochemical features. In addition, we reviewed and analyzed data from the Texas Cancer Registry between the years 1995 and 2016 in order to provide insight into the demography and epidemiology of BL originating in the colon.

This paper reports a 69-year-old male who presented with a history of irritable bowel syndrome, was diagnosed with BL in the colon, and subsequently developed abdominal compartment syndrome. Biopsies derived from the colon tumor at three different sites showed infiltrating malignant lymphoma of the lamina propria. Immunohistochemistry stains of lymphoma cells were positive for CD20, CD79a, CD10, MUM1, BCL6, C-MYC, and negative for BCL2, cyclin D1, CD5, and CD3. Ki-67 demonstrated a high proliferative index of 100%. Forty-nine cases of primary BL of the colon were reported to the Texas Cancer Registry between 1995 and 2016. The unadjusted incidence of BL originating in the colon in persons 18 years old and over was 1.32 per 10 million, and the majority of the cases involved non-Hispanic white males with cecum being the most common primary site. BL is a rapidly growing malignancy, hence, reporting cases of BL and its presenting symptoms can improve assessment and management. Our analysis from the Texas Cancer Registry further supports the rarity of primary sporadic BL in the colon.

## Introduction

Burkitt’s lymphoma (BL) is a highly aggressive non-Hodgkin’s B-cell lymphoma (NHL) recognized by three epidemiological forms: endemic, sporadic, and immunodeficiency-associated. Endemic BL cases are seen in equatorial Africa among children presenting with facial tumors [1,2]. Immunodeficiency-associated BL has been reported in AIDS cases and transplant recipients on immunosuppressors [1-3]. The sporadic form of BL is seen in the United States and Western Europe and comprises <1% of adult NHL in the United States. Sporadic BL primarily involves the stomach, cecum, and distal ileum, and mimics various gastrointestinal (GI) symptoms (e.g. acute appendicitis or intussusception) [3].

Though there are reports of extranodal spread, primary NHL of the colon is an extremely rare case, encompassing only 0.1-0.5% of all malignant tumors of the colon [1]. Sporadic BL may be misdiagnosed due to its rarity and non-specific GI presentation. We, therefore, report one case of primary sporadic BL of the colon with presenting features of irritable bowel syndrome (IBS) and subsequent development of abdominal compartment syndrome (ACS). Information from the Texas Cancer Registry [4] was reviewed to calculate the unadjusted incidence of Burkitt’s lymphoma originating in the colon in persons over the age of 18 years from 1995-2016.

## Case presentation

A 69-year-old Hispanic male presented with a five-month history of alternating diarrhea and constipation, progressive abdominal swelling with pressure-like symptoms, worsening bulging of bilateral inguinal hernias, and 5-kg weight loss. His medications consisted of an angiotensin-converting-enzyme inhibitor for the management of hypertension, a proton pump inhibitor for the management of gastroesophageal reflux disease, and an alpha-1 blocker for medical management of benign prostate hypertrophy. His family history was significant for two sisters with unidentified malignancies. He had a 20-pack-year smoking history and no history of alcohol or illicit drug abuse. The patient had a colonoscopy five years ago that showed four benign polyps. Physical examination revealed a distended abdomen with normal bowel sounds and bilateral bulging, but reducible inguinal hernias. Rectal examination revealed medium-sized external hemorrhoids with no melena or hematochezia. An initial laboratory workup revealed a white blood count of 11260/mm^3^, hemoglobin of 14.6 g/dL, hematocrit of 41.7%, and a platelet count of 311000/mm^3^. The biochemical profile was normal with aspartate aminotransferase of 72 U/L, alkaline phosphatase of 79 U/L, alanine aminotransferase of 37 U/L, and carcinoembryonic antigen of 1.3 ng/mL. The patient was negative for HIV and had no noted prior pesticide or chemical exposures.

**Figure 1:**
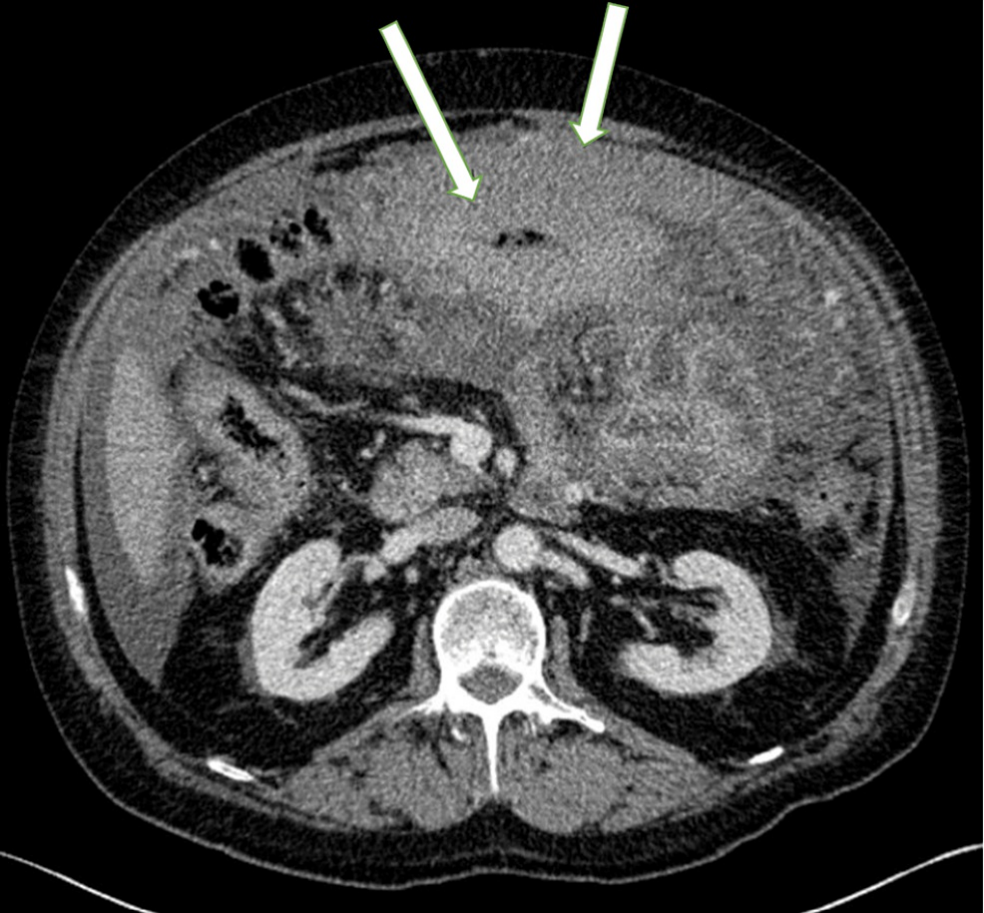
CT scan of the distal transverse colon CT scan findings show short segment circumferential wall thickening at the distal transverse colon which suggests luminal narrowing (see arrows).

Computed tomography (CT) scans of the abdomen and pelvis (Figure 1) in the emergency department showed moderate volume ascitic fluid with omental caking of the anterior parietal peritoneum. There was associated short segment circumferential wall thickening of the distal transverse colon suggesting luminal narrowing. 

**Figure 2:**
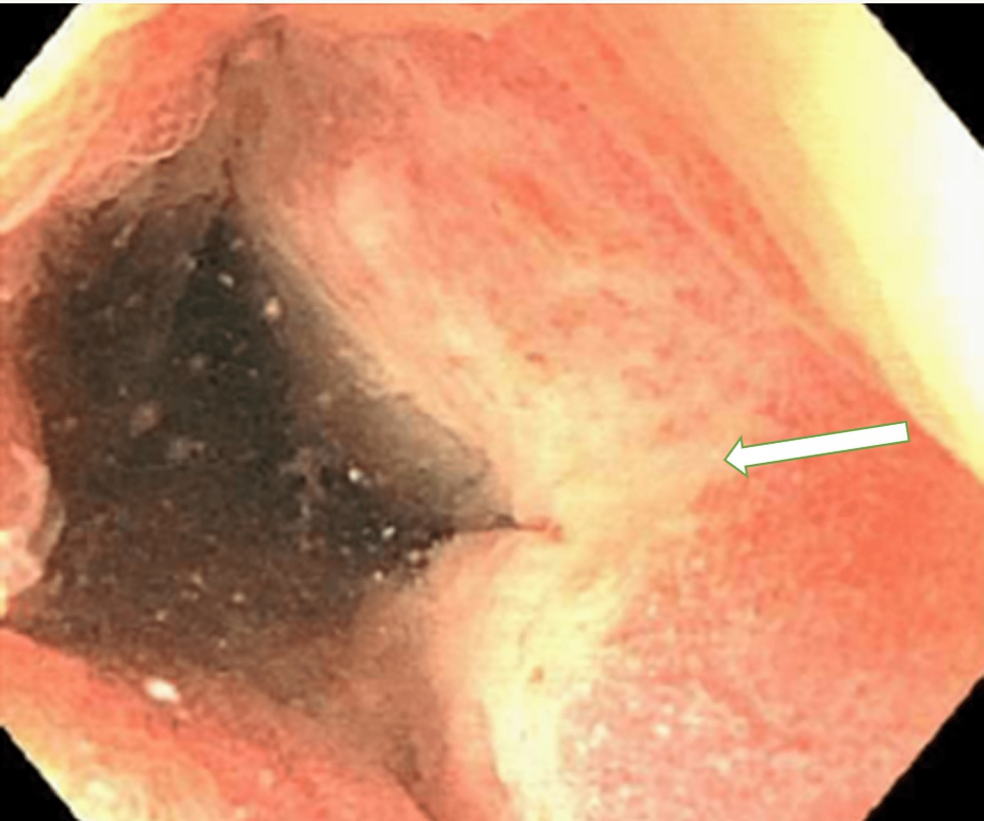
Colonoscopy of the distal transverse colon with abnormal mucosa Colonoscopy findings show congested, erythematous, friable, malignant-appearing, nodular, and ulcerated mucosa (see arrow) of the distal transverse colon.

**Figure 3:**
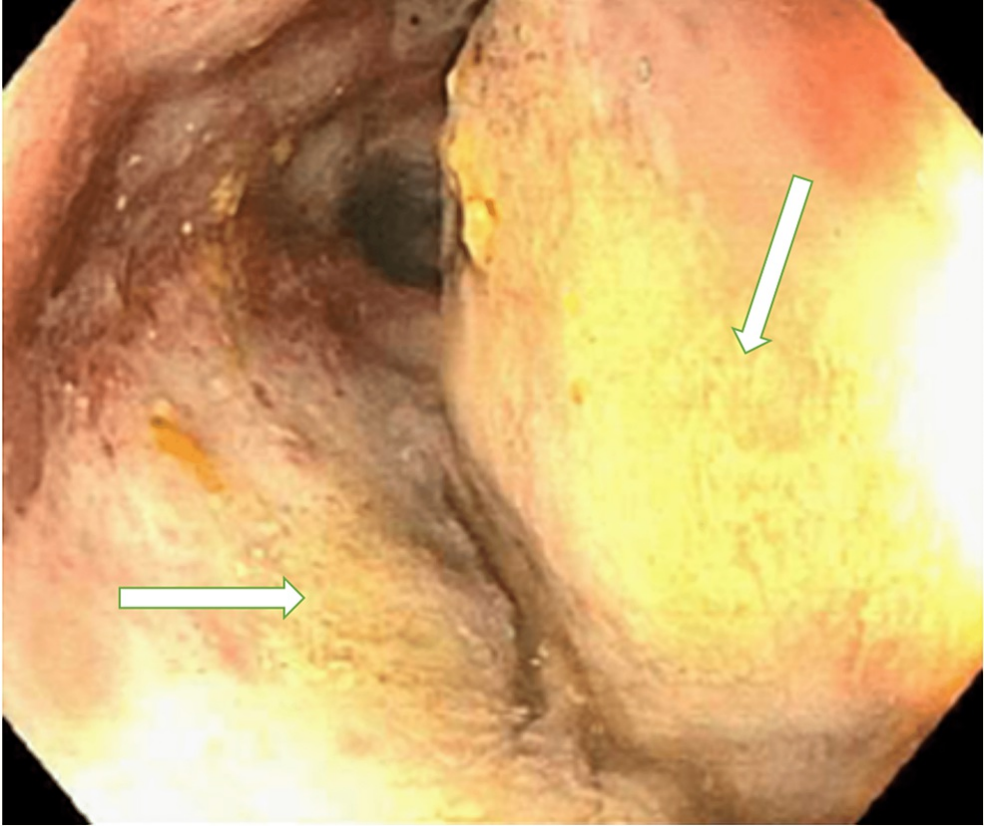
Colonoscopy of the distal transverse colon with ulcerated mucosa Colonoscopy findings show congested, erythematous, friable, malignant-appearing, nodular, and ulcerated mucosa (see arrows) of the distal transverse colon.

**Figure 4:**
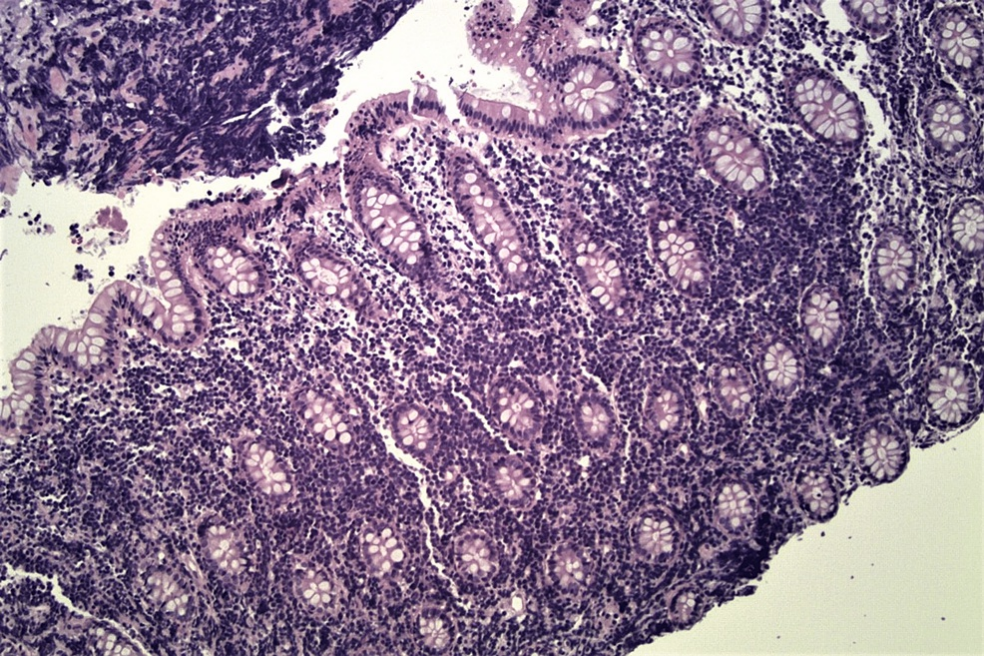
Histologic section of colonic mucosa with H&E stain (original magnification, x 200). Colonic mucosa shows expansion of the lamina propria by lymphoma cells.

**Figure 5:**
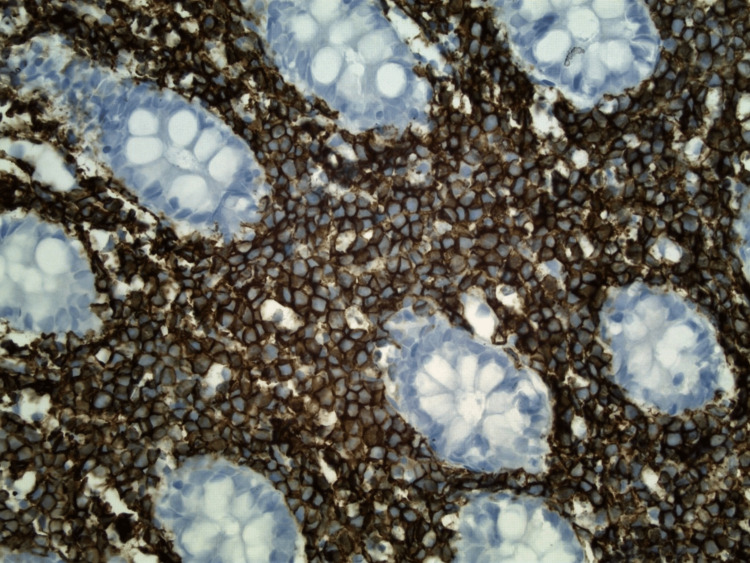
Histologic section of colonic mucosa with CD20 immunohistochemical stain (original magnification, x 400) The lymphoma cells show diffuse and intense staining with C20 immunohistochemical stain (brown).

**Figure 6:**
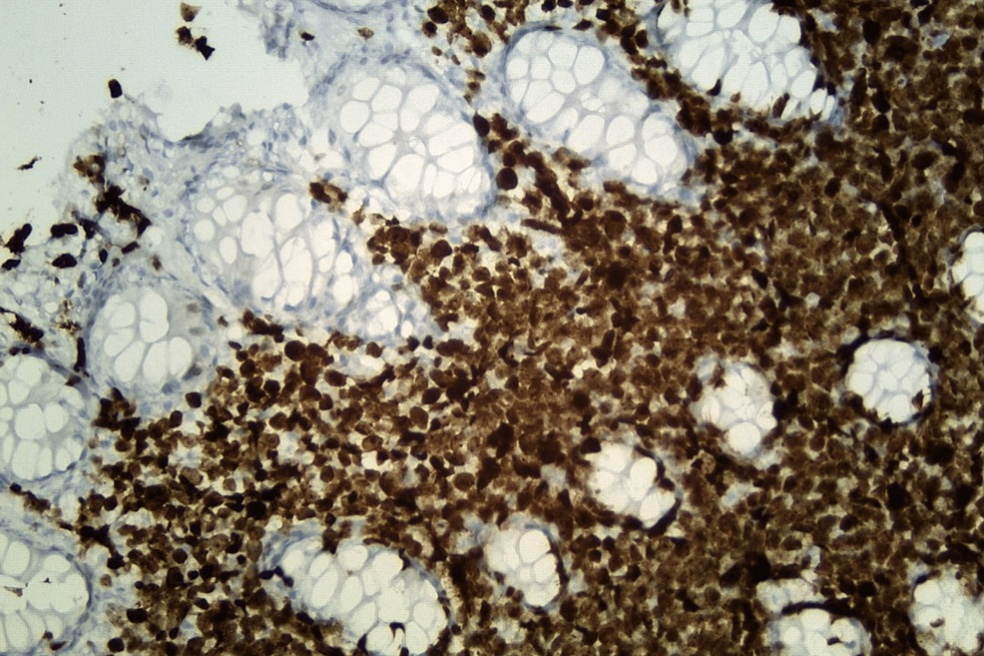
Histological section of colonic mucosa with Ki-67 immunohistochemical stain (original magnification, x 400). The lymphoma cells show high proliferative index by Ki-67 immunohistochemical stain (brown).

A colonoscopy revealed erythematous and friable malignant-appearing nodular and ulcerated mucosa in the distal transverse colon extending between 60-70 cm from the anal verge (Figure 2, 3). Biopsies derived from the colon tumor at 60 cm, 65cm, and 70 cm showed infiltrating malignant lymphoma involving the lamina propria (Figure 4). The lymphoma cells were positive for CD20, CD79a, CD10, MUM1, and BCL6 immunostains (Figure 5). The lymphoma cells were positive for C-MYC and negative for BCL2, cyclin D1, CD5, and CD3. Ki-67 demonstrated a high proliferative index of 100% (Figure 6). The morphologic and immunohistochemical features were consistent with the diagnosis of BL Esophagogastroduodenoscopy and biopsies of the distal esophagus, gastroesophageal junction, gastric antrum, and gastric body were only significant for gastritis and reflux esophagitis. 

During the hospital course, the patient developed acute kidney injury secondary to increasing ascites from the BL with intra-abdominal pressure reaching 20 mmHg. The treatment plan was discussed with the patient and family members who declined further management and opted for comfort measures only (CMO). The patient was discharged home with hospice.

## Discussion

The Texas Cancer Registry (Department of State Health Services, Austin, Texas) provided demographic and epidemiological information on reported cases of BL originating in the colon. Cases were Texas residents between the years of 1995-2016. The primary site of the cancer and its morphology in the Texas Cancer Registry had been coded using the International Classification of Diseases for Oncology Third Edition (ICD-O-3). The primary site of colon is coded as C18.0-C18.9. All of the cases that were reviewed by the study team contained a primary site code between C18.0-C18.9. For our analysis, BL was defined as a morphology code of 9687/3.

The unadjusted incidence of BL originating in the colon in adults aged 18 years or older in Texas from the period of 1995 through 2016 was calculated by dividing the number of reported cases during this time period by the total population of adults aged 18 and over from the time period of 1995-2016 (Texas Department of State Health Services, 2019) [4]. The incidence was reported as the risk per 10 million persons.

The query of the Texas cancer registry returned a total of 49 cases of Burkitt’s lymphoma originating in the colon. The unadjusted incidence of Burkitt’s lymphoma originating in the colon in persons aged 18 and over was 1.32 per 10 million. There were 18 additional reported cases. However, these cases were excluded from our analysis due to the persons being under the age of 18. 

Of the included cases, 30 were reported as non-Hispanic whites (61.2%), 17 were reported as Hispanic white (34.7%), and two were reported as African American (4.1%) (Table 1). Forty of the cases were male (81.6%) while the remaining 9 were female (18.4%). When looking at specific sites within the colon, 20 were located in the cecum (40.8%), seven in the colon not otherwise specified (NOS) (14.3%), eight in the ascending colon (16.3%), four in the sigmoid colon (8.2%), four in the setting of an overlapping lesion (8.2%), three in the hepatic flexure (6.1%), two in the appendix (4%), and one in the descending colon (2%).

**Table 1:** Cases of Burkitt’s lymphoma originating in colon 1995-2016 Code: Texas Cancer Registry coded using the International Classification of Diseases for Oncology Third Edition NOS: Not otherwise specified N: Number

Primary Site (Code)	N	Age Mean in years (Range)	Male (%)	White (%)	Hispanic (%)
Appendix (18.1)	2	31 (18-43)	100	50	50
Ascending Colon (18.2)	7	47 (19-69)	86	71	14
Cecum (18.0)	20	49 (20-85)	80	65	35
Colon NOS (18.9)	7	57 (40-75)	86	71	29
Descending Colon (18.6)	1	48	100	100	0
Hepatic Flexure (18.3)	3	37 (33-43)	100	0	66
Sigmoid Colon (18.7)	4	72 (51-85)	40	60	40
Overlapping Lesion of Colon (18.8)	4	55 (18-72)	100	50	50

Sporadic BL accounts for 1-2% of NHL in all adults in the United States and Western Europe [3]. While primary NHL of the GI tract is commonly found in the stomach (50-60%) and small intestine (30%), only 10% of all cases are localized to the large bowel and rectum [5]. Therefore, primary lymphoma of the colon is very rare, comprising only 0.1-0.5% of all colonic malignancies [5,6]. Published reports of sporadic BL present evidence of its GI involvement, but very few reports have described primary colonic findings in the immunocompetent adult population [1,2,5].

The clinical symptoms of sporadic BL are various, including abdominal pain, nausea, vomiting, bowel obstruction, ascites, abdominal distention, melena, and hematochezia, and may also mimic signs of acute appendicitis or intussusception [1,2,6-8]. Other involvement of sporadic BL includes bone marrow, central nervous system, kidney, testis, ovary, and breast [6,9,10], and may present with symptoms related to these systems. In comparison, endemic BL is commonly observed in African children of four to seven years, with frequent involvement of the jaw and kidneys [2,5,10]. The combination of the symptoms mentioned above can lead to false diagnoses and delayed treatment. Hence, reporting cases of BL and its presenting symptoms becomes imperative for better assessment and management. Our patient presented with a history of IBS and was found to have a rare finding of BL in the colon, and subsequently developed Abdominal Compartment Syndrome.

The diagnosis of BL is based on pathological evaluation. This includes evaluation of B-cell-associated antigens and germinal center-associated markers. BL cells express surface IgM, CD10, CD19, CD20, CD22, and CD79A [3,6,8], and are negative for, CD5, CD23, BCL2, and TdT [6,8]. Expression of BCL-6 and CD10 suggest germinal center origin for BL [3]. In addition, BL cells express a high proliferation rate with the Ki-67 close to 100 percent [8]. Expression of CD21, the Epstein-Barr virus/C3d receptor, is associated with endemic BL, whereas the vast majority of non-endemic BL in non-immunocompromised patients lack this expression pattern [6].

The prognosis of patients with BL has significantly changed with the advent of short, intensive chemotherapy. Several agents, including bleomycin, cyclophosphamide, vincristine, doxorubicin, and high-dose methotrexate, provide excellent response rates [11] achieving a complete response rate of 85%, with a five-year disease-free survival rate of 60%[5,9,10,12].

Between the years of 1995-2016, only 49 cases of Burkitt’s lymphoma originating in the colon in persons over 18 were reported in Texas, with an unadjusted incidence of only 1.32 per 10 million. The majority of reported cases originated in non-Hispanic whites (61.2%) while the least number of reported cases were in African Americans (4.1%). The vast majority of cases were male, comprising 81.6% of the reported cases. When looking at specific origins within the colon, the majority were located in the cecum (40.8%), with 14.3% of the samples being within the colon but not otherwise specified. Only one case was reported to have originated in the descending colon.

## Conclusions

BL is a rare malignancy in adults that infrequently involves the GI tract. We report a patient who presented with involvement of the colon. Our case report represents the importance of including BL in the differential diagnoses of patients with nonspecific GI symptoms, including changes in bowel habits and abdominal distension. BL is a rapidly growing malignancy, but prompt diagnosis and treatment can lead to better outcomes. In addition to our reported case, a review of the Texas cancer registry demonstrates how rare BL originating in the colon is. It also gives us an insight into the incidence and demographics of the affected individuals. We found that the majority of the cases reported in Texas between the years of 1995-2016 were non-Hispanic white males, with cecum being the most common primary site.
